# APC/C^Cdh1^ Targets Brain-Specific Kinase 2 (BRSK2) for Degradation via the Ubiquitin-Proteasome Pathway

**DOI:** 10.1371/journal.pone.0045932

**Published:** 2012-09-21

**Authors:** Ruwei Li, Bo Wan, Jun Zhou, Yingli Wang, Ting Luo, Xiuting Gu, Fang Chen, Long Yu

**Affiliations:** State Key Laboratory of Genetic Engineering, School of Life Sciences, Fudan University, Shanghai, People’s Republic of China; University of Minnesota, United States of America

## Abstract

Studies of brain-specific kinase 2 (BRSK2), an AMP-activated protein kinase (AMPK)-related kinase, and its homologs suggest that they are multifunctional regulators of cell-cycle progression. BRSK2, which contains a ubiquitin-associated (UBA) domain, is polyubiquitinated in cells. However, the regulatory mechanisms and exact biological function of BRSK2 remain unclear. Herein, we show that BRSK2 co-localizes with the centrosomes during mitosis. We also demonstrate that BRSK2 protein levels fluctuate during the cell cycle, peaking during mitosis and declining in G1 phase. Furthermore, Cdh1, rather than Cdc20, promotes the degradation of BRSK2 *in vivo*. Consistent with this finding, knock-down of endogenous Cdh1 blocks BRSK2 degradation during the G1 phase. The conserved KEN box of BRSK2 is required for anaphase-promoting complex/cyclosome-Cdh1 (APC/C^Cdh1^)-dependent degradation. Additionally, overexpression of either BRSK2(WT) or BRSK2(ΔKEN) increases the percentage of cells in G2/M. Thus, our results provide the first evidence that BRSK2 regulates cell-cycle progression controlled by APC/C^Cdh1^ through the ubiquitin-proteasome pathway.

## Introduction

Brain-specific kinase 2 (BRSK2) was initially identified through sequence homology with the protein kinase domain of AMPK and is thus classified as an AMPK-related kinase [1]. Northern and Western blot analyses have shown that BRSK2 is expressed at high levels in the brain and at lower levels in the testis and pancreas [2]. Recently, Chen X et al found that BRSK2 is also expressed in some tumor cell lines, such as HeLa and Panc-1 [3]. Studies of mice lacking both BRSK1 and BRSK2 (BRSK1/2) confirmed a role for these kinases in regulating neuronal polarity, presynaptic vesicle clustering and axon termination [4]–[7]. A recent study indicated that BRSK1/2 acts downstream of LKB1 in the establishment of axon specification [8]–[10]. Intriguingly, a role for BRSK2 and its homologs in other molecular regulatory functions, especially cell cycle regulation, was reported recently. There are two important cell cycle checkpoint kinases, Wee1 and Cdc25, which regulate mitotic entry in dividing cells. Cdr2, the BRSK1 homolog in fission yeast, was reported to regulate mitosis through an association with the Wee1 protein kinase [11], [12]. Further studies in human cells showed that human BRSK1 (SAD1) enables inhibition of Cdk1 by phosphorylating Wee1A and Cdc25B/C on the second Ser of an S-X-S motif [13]. A recent report also provided evidence that BRSK2 (SADA) regulates the activity of Wee1 through phosphorylation [14]. γ-tubulin is an essential component of the centrosome, the microtubule organizing center that tightly regulates the cell cycle. Hsl1, the yeast homolog of BRSK2 (human sequence similar to yeast-12, HUSSY-12) [15], phosphorylates γ-tubulin *in vitro* [16]; another investigation reported that BRSK1 (SADB) phosphorylates γ-tubulin on Ser 131 to control centrosome homeostasis [17]. Thus, these studies imply that BRSK2 plays an important role in the regulation of cell cycle progression.

Although many roles for BRSK2 in regulating the cell cycle have been explored, the explicit regulatory mechanisms underlying BRSK2 are still unclear. Recently, the first *in vivo* evidence for polyubiquitination of BRSK2 and several other AMPK-related kinases (AMPK-RKs) by atypical lysine29- and lysine33- linked chains was reported [18]. AMPK-RKs are the only kinases in the human genome that contain a ubiquitin-associated (UBA) domain, which is located immediately C-terminal to their catalytic domain [1]. The UBA domain interacts with the catalytic domain to produce a conformation that can be phosphorylated and activated by LKB1 [19]. An investigation of the polyubiquitination of AMPK-RKs showed that the deubiquitinating enzyme USP9X (ubiquitin specific protease-9) modulates the deubiquitination of NUAK1 (AMPK-related kinase 5) and MARK4 (microtubule-affinity-regulating kinase 4) in cells [18]. One study identified an E3 ubiquitin ligase, Cidea (cell death inducing DFFA-like effector a), that mediates the ubiquitination and degradation of AMPK [20]. Very recently, we also reported that BRSK2 undergoes degradation via the ubiquitin-proteasome pathway *in vivo* [21]. Thus, previous studies indicated that the modulation of BRSK2 protein levels can partly account for the regulation of its kinase activity during the cell cycle.

The ubiquitin-proteasome system is a commonly used mechanism in eukaryotic cells to regulate biological transitions through protein destruction [22], [23]. This system requires three enzymes: a ubiquitin-activating enzyme (E1), a conjugating enzyme (E2), and a ubiquitin ligase (E3). Ubiquitin is activated by an E1, and the activated ubiquitin is then transferred to an E2 and subsequently ligated to the substrate protein via an E3. Finally, the polyubiquitinated protein is degraded by the 26S proteasome [22].

The anaphase-promoting complex/cyclosome (APC/C), a multisubunit E3 ubiquitin ligase, plays a central role in regulating mitotic entry and progress as well as DNA replication by targeting a wide variety of substrates, such as Cdc6, Cyclin B and securin [24]–[26]. APC/C activity must be tightly controlled to prevent unscheduled substrate degradation. In addition to the mitosis-specific phosphorylation of APC/C subunits, the activation of APC/C requires the binding of Fizzy family proteins Cdc20 and Cdh1. Cdc20 directly binds to and activates APC/C at the onset of anaphase, whereas Cdh1 replaces Cdc20 during late mitosis and remains associated with APC/C until late G1 [27]. The substrates selected by APC/C for destruction contain specific sequences called APC/C degrons [28]. Several APC/C degrons have been characterized, including the D box (RXXLXXXN, X indicates any amino acid) and the KEN box. The D box can be recognized by either APC/C^Cdh1^ or APC/C^Cdc20^, while the substrates only containing the KEN box are recognized and ubiquitinated by APC/C^Cdh1^ [29], [30]. Ubiquitin- and proteasome-dependent degradation is one of the key pathways ensuring proper cell cycle progression.

In this paper, we demonstrate that BRSK2 is a novel substrate of APC/C^Cdh1^ ubiquitin E3 ligase complex and undergoes proteolysis from anaphase through G1 phase, allowing for proper cell cycle progression.

## Materials and Methods

### Cell Lines, Synchronization and Drug Treatment

HeLa, HEK293T and H1299 cells were obtained from ATCC. HeLa and HEK293T cells were cultured in Dulbecco’s modified Eagle’s medium (DMEM, Invitrogen) plus 10% FBS (PAA) at 37°C with 5% CO_2_. H1299 cells were maintained in RPMI 1640 with 10% FBS. The cells were synchronized at the G1/S transition using a double thymidine block. Briefly, the HeLa cells were treated with 2 mM thymidine (Sigma) for 22.5 h, washed three times with PBS and cultured in fresh medium for 10 h. The cells were treated again with thymidine for 16 h and then released from the block by washing with PBS and replacing the medium with fresh DMEM plus 10% FBS. The HeLa cells were synchronized at prometaphase using a thymidine-nocodazole block. Briefly, the cells were incubated with 2 mM thymidine for 22.5 h, washed three times with PBS and cultured in fresh medium for 3 h. The cells were then treated with 500 ng/mL nocodazole (Sigma) for 12 h. The protein synthesis inhibitor cycloheximide (Sigma) was dissolved in H_2_O and added to the culture medium at a concentration of 10 ng/µL. The proteasome inhibitors MG132 and Lactacystin were used at a final concentration of 20 µM and 10 µM, respectively.

### Mammalian Expression Plasmids and Transfection

PCMV-HA-BRSK2 was generated by inserting the full-length BRSK2 cDNA into the SfiI and XhoI sites of PCMV-HA vector (Clonetech). Myc-Cdh1 and Myc-Cdc20 were kind gifts from Zee-Fen Chang (Graduate Institute of Biochemistry and Molecular Biology, College of Medicine, National Taiwan University, Taipei, Taiwan, Republic of China). BRSK2 D box and KEN mutants were generated using a KOD-Plus Mutagenesis Kit (Toyobo Japan) according to the manufacturer’s instructions. All of the constructs were confirmed by sequencing. Transient cell transfections were performed using Lipofectamine 2000 (Invitrogen) according to the manufacturer’s instructions.

### Cell Cycle Analysis

Cellular DNA content was assessed by propidium iodide (PI) staining as follows: cells were harvested, washed with PBS, fixed in 70% ice-cold ethanol overnight at 4°C, washed again with PBS, incubated with 100 µg/mL RNaseA (Sigma) for 30 min at 37°C, stained with 50 µg/mL PI (Sigma) for 30 min at room temperature and then analyzed by flow cytometry. The percentage of cells in each cell cycle phase was quantified using the ModFit program.

### Western Blot Analysis and Immunoprecipitations

For Western blot analysis, cells were washed once in PBS, resuspended in cell lysis buffer (Cell Signaling Technology) with a protease inhibitor mixture containing PMSF and Cocktail, and then incubated on ice for 30 min. The lysates were then cleared by centrifugation at 12,000 rpm for 5 min. The total protein was boiled for 8 min with SDS sample buffer.

Equal amounts of protein sample were then separated on SDS polyacrylamide gels of the indicated concentrations and transferred to nitrocellulose membranes (Whatman). The membranes were then blocked for 1 h at room temperature in 5% skim milk with 0.5% tween-TBS (TBST) and then probed with primary antibodies overnight at 4°C in TBST containing 3% skim milk. After being washed 3 times for 7 min with TBST, the membranes were incubated at room temperature for 1 h with the indicated secondary antibodies diluted in 3% skim milk. Following 3 7 min washes in TBST, the proteins were visualized by ECL (Santa Cruz Biotechnology).

For the immunoprecipitations, cells were lysed in cell lysis buffer (20 mM Tris-HCl pH 7.5, 150 mM NaCl, 1% Triton, 1 mM EGTA, 1 mM Na_2_EDTA, 2.5 mM sodium pyrophosphate, 1 mMβ-glycerophosphate, 1 mM Na_3_VO_4_ and 1 µg/ml leupeptin) for 1 h at 4°C and then centrifuged at 12,000 rpm for 20 min. The supernatant was precleared with protein A/G, followed by incubation with 2 µL of primary antibody overnight at 4°C. Thirty microliters of a protein A/G bead slurry (GE Healthcare) was added for an additional hour prior to an extensive wash in cell lysis buffer. After being washed, the pellets were boiled in SDS sample buffer for 5 min, and the immunoprecipitates were analyzed by Western blotting as described above.

### Immunofluorescence Assay

HeLa cells cultured on coverslips were fixed for 10 min in 4% paraformaldehyde and permeabilized with 0.2% Triton X-100 for 5 min at room temperature. The coverslips were blocked with 10% normal horse serum plus 1% BSA for 1 h. The cells were co-stained with rabbit anti-BRSK2 antibody (1∶400) and mouse anti-γ-tubulin antibody (1∶400), washed 3 times for 7 min in TBST and then incubated with Alexa 488 (green fluorescence)-conjugated goat anti-rabbit secondary antibody and Alexa 555 (red fluorescence)-conjugated goat anti-mouse secondary antibody for 1 h. The nuclei were stained with DAPI (1∶1,000) for 5 min. Confocal images were captured using an Olympus FluoView FV1000 confocal fluorescence microscope.

### Antibodies

The following primary antibodies were used: anti-BRSK2 (1∶1,000, rabbit polyclonal antibody prepared in our laboratory [3]), anti-HA (1∶1,000, Sigma), anti-Flag M2 (1∶2,000, Sigma), anti-Myc (1∶3,000, Santa Cruz Biotechnology), anti-Cdh1 (1∶1,000, Sigma), anti-cyclin B1 (1∶1,000, Santa Cruz Biotechnology), anti-Aurora A (1∶1,000, Santa Cruz Biotechnology), anti-Aurora B (1∶1,000, BD Biosciences), anti-p-Histone H3(Ser10) (1∶1,000, Sigma), anti-β-tubulin (1∶5,000, Sigma), anti-γ-tubulin (1∶500, Santa Cruz Biotechnology), anti-GFP (1∶500, Santa Cruz Biotechnology) and anti-β-actin (1∶5,000, Sigma).

### RNA Interference

siRNA duplexes were synthesized by Genepharma (Shanghai, China). The two siRNA oligonucleotide sequences for Cdh1 were 5′-(UGA GAA GUC UCC CAG UCA G) dTdT-3′ and 5′-(AAU GAG AAG UCU CCC AGU CAG) dTdT-3′. The negative control sequence was 5′-(UUC UCC GAA CGU GUC ACG U) dTdT-3′. For siRNA transfection, cells were transfected with 25 nM siRNA using Lipofectamine 2000 according to the manufacturer’s instructions.

### *In Vivo* Ubiquitination Experiments

The *in vivo* ubiquitination assay was similar to the immunoprecipitation procedure. HEK293T cells were co-transfected with plasmids encoding either HA-BRSK2, Flag-Ub or Myc-Cdh1 with or without MG132 treatment. Twenty-four hours post-transfection, the cells were harvested, and HA-BRSK2 was immunoprecipitated using anti-HA antibody. Both the immuonoprecipitates and whole-cell lysates were blotted with anti-FLAG M2 antibody for Flag-tagged ubiquitin.

### RNA Preparation and Quantitative Real-time PCR

Total RNA was extracted from HEK293T cells using Trizol (Invitrogen) according to the manufacturer’s instructions. cDNA was reversed-transcribed using the Superscript RT kit (Toyobo, Japan) according to the manufacturer’s instructions. Each PCR was carried out in triplicate in a 10 µL volume using SYBR Green PCR Master Mix (Toyobo) with a Roche Lightcycler 480 II Real-Time PCR System using the following conditions: 5 min at 95°C for initial denaturing, followed by 40 cycles of 95°C for 10 s and 62°C for 30 s The results were analyzed with Lightcycler SW 1.5 software. All quantitations were normalized to the level of the endogenous control GAPDH. Each reaction was performed in triplicate from at least two independent experiments. The primer sequences for BRSK2 and GAPDH were 5′-GCTGGCGAAGAAGTCCTGGT-3′ (BRSK2 forward), 5′-CGT GGC CTT GTA CTC GGC-3′ (BRSK2 reverse); 5′-AGG GCT GCT TTT AAC TCT GGT-3′ (GAPDH forward), 5′-CCC CAC TTG ATT TTG GAG GGA-3′ (GAPDH reverse).

### Statistical Analysis

The data in this study were expressed as the mean ± S.D. from three independent experiments.

## Results

### BRSK2 Localizes to Centrosomes during Mitosis

**Figure 1:**
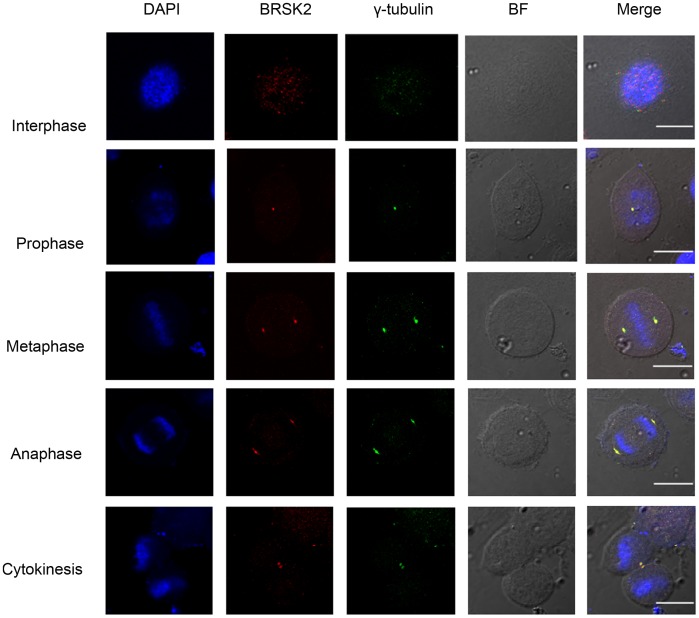
BRSK2 co-localizes with the centrosomes during mitosis. Synchronously growing HeLa cells were fixed and stained as described in “Materials and Methods”. This figure shows representative images of dividing cells captured in different channels for BRSK2 (red), γ-tubulin (green), and DAPI (blue). All images were captured with an Olympus FluoView FV1000 confocal fluorescence microscope and a 60x oil immersion objective lens. BF: bright field. Scale bar, 10 µm.

Recent evidence showed that the BRSK2 homologue BRSK1 (SADB), whose activity fluctuates during the cell cycle, localizes to centrosomes and controls centrosome duplication [17]. However, the subcellular localization of BRSK2 during the cell cycle is still unknown. Similar to the BRSK1 localization, the images of subcellular localization showed that areas corresponding to the patterns of centrosomes were stained with BRSK2 in the mitosis phase. Thus, we sought to confirm the centrosomal localization of BRSK2 through co-localization analyses of BRSK2 with γ-tubulin, a specific marker for centrosomes. Co-localization between γ-tubulin and BRSK2 was not observed during interphase in HeLa cells (Fig. 1). γ-tubulin co-localized with BRSK2 in the duplicated centrosomes as the cells progressed through mitosis from prophase to cytokinesis. This finding suggests that BRSK2 is a centrosome-localized protein.

### BRSK2 Protein Levels Fluctuate during the Cell Cycle

**Figure 2:**
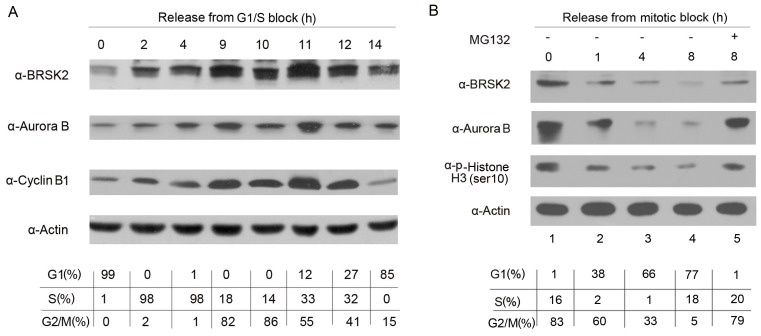
BRSK2 protein levels fluctuate during the cell cycle. (A) HeLa cells were arrested at the G1-S boundary by a double-thymidine block, released into fresh medium, and harvested at the indicated time points. Cell lysates were probed by Western blot (WB) with BRSK2, Aurora B, Cyclin B1, and β-actin antibodies. β-actin served as a loading control. (B) HeLa cells were arrested at prometaphase by a thymidine-nocodazole treatment, released into fresh medium, and harvested at the indicated time points. Cell lysates were probed by Western blot (WB) with BRSK2, Aurora B, p-Histone H3(Ser10), and β-actin antibodies. Cells were treated with MG132 (20 µM) for 8 h during the mitotic exit phase. Cell cycle stages in (A) and (B) were determined by flow cytometry and are indicated in the figure.

The abundance of endogenous BRSK2 was examined in HeLa cells at various points throughout the cell cycle. HeLa cells arrested at the G1/S boundary by a double-thymidine block were released into fresh medium to allow progression through the cell cycle synchronously from the G1/S boundary to mitosis until the next G1 phase. Whole-cell extracts were prepared, and the protein levels were analyzed by Western blotting. The BRSK2 protein levels were lowest at the G1/S boundary but gradually increased as the cells progressed into G2 phase (Fig. 2A). The BRSK2 levels peaked 11 hours after release, a time at which the majority of cells had entered mitosis. As the cells exited mitosis and entered G1, BRSK2 rapidly decreased to very low levels. Notably, the BRSK2 levels behaved similarly to the levels of Aurora B and cyclin B1 (Fig. 2A), two known mitotic substrates of APC/C [27], [31]. To further confirm the kinetics of BRSK2 downregulation at mitotic exit, we next synchronized HeLa cells at prometaphase using a thymidine-nocodazole block followed by release into fresh medium, which allowed the cells to synchronously exit mitosis and enter G1. We observed that the BRSK2 levels declined as the cells completed mitosis and that the BRSK2 levels again mimicked levels of Aurora B (Fig. 2B). The levels of p-Histone H3(Ser10) protein indicated the stages during cell cycle progression. Thus, BRSK2 protein levels fluctuated during the cell cycle in a manner similar to known APC/C substrates.

### BRSK2 is Degraded via the Ubiquitin-proteasome Pathway

**Figure 3:**
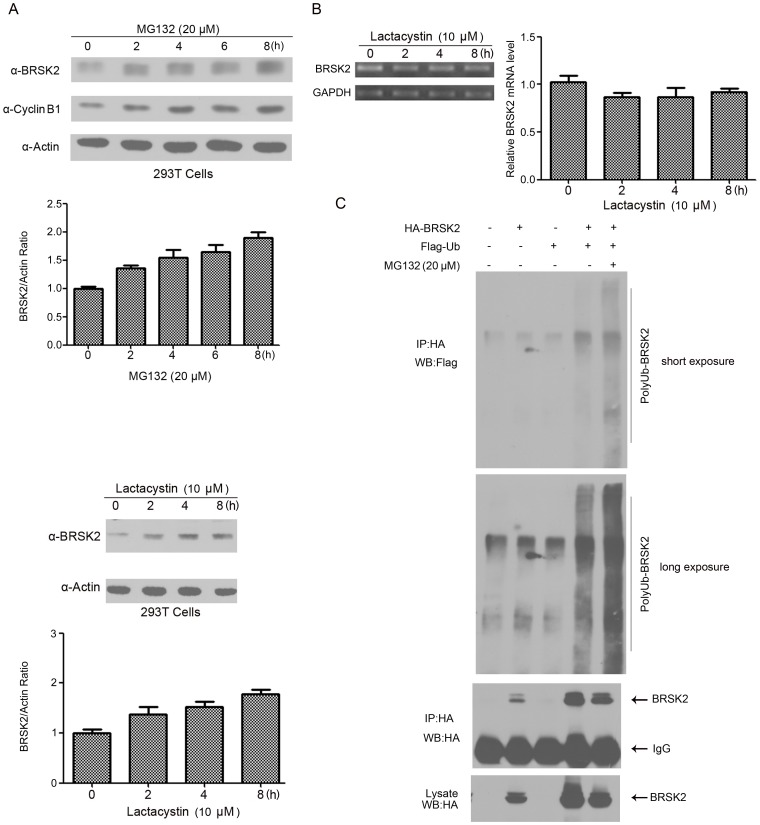
BRSK2 stability is controlled by the ubiquitin-proteasome pathway. (A) HEK293T cells were treated with each 20 µM MG132 or 10 µM lactacystin for the indicated times. Equal amounts of total cell lysates were subjected to Western blot (WB) analysis using antibodies against BRSK2, Cyclin B1 and β-actin. The mean values (± S.D.) of three independent experiments are shown. (B) qRT-PCR measurements of BRSK2 mRNA levels in HEK293T cells after treatment with lactacystin at the indicated time points. GAPDH mRNA levels were used for normalization. (C) HEK293T cells were transfected with (–) or without (+) HA-BRSK2 or Flag-Ub as indicated. BRSK2 was immunoprecipitated (IP) with anti-HA antibody, and the immunocomplexes were analyzed by SDS-PAGE and Western blotting (WB) analysis using anti-HA and anti-Flag M2 antibodies.

Consistent with evidence that the majority of, if not all, mitotic protein degradation is mediated by the proteasomal pathway, we recently reported that BRSK2 may be degraded in this pathway *in vivo* [21]. To further investigate this possibility, we treated HEK293T cells with the proteasome inhibitor MG132 for 0, 2, 4, 6 and 8 h and determined the levels of endogenous BRSK2 protein by Western blotting. As shown in the upper panels of Fig. 3A, MG132 treatment led to an increase in the BRSK2 protein levels in HEK293T cells. MG132 treatment during the release from prometaphase arrest inhibited BRSK2 degradation and caused a concomitant stabilization of Aurora B (Fig. 2B, lanes 4 and 5). Because MG132 also inhibits non-proteasomal enzymes, we repeated the experiment using another proteasome inhibitor, lactacystin, which only targets the proteasome [32]. Lactacystin treatment led to a greater increase in BRSK2 protein levels than that caused by MG132 treatment in HEK293T cells (Fig. 3A, lower panels). To exclude the possibility that the higher protein levels might have resulted from the upregulation of transcription, we performed qRT-PCR to measure BRSK2 mRNA levels in HEK293T cells following lactacystin treatment. As shown in Fig. 3B, lactacystin had little effect on the mRNA level of BRSK2. Together, these results suggested that BRSK2 stability might be regulated by the proteasomal pathway.

To further explore whether BRSK2 could be polyubiquitinylated *in vivo*, we transfected HEK293T cells with expression vectors for Flag-Ub and HA-BRSK2. Cell extracts were prepared for immunoprecipitation with anti-HA antibody and followed by Western blotting analysis using anti-Flag M2 antibody (Fig. 3C). Our results clearly demonstrated the existence of Flag-tagged polyubiquitinylated forms of BRSK2 in cells co-expressing HA-BRSK2 and Flag-Ub. Treatment of these transfected cells with MG132 enhanced BRSK2 polyubiquitinylation. These data provided clear evidence that the ubiquitin-protesome pathway controls the destruction of BRSK2.

### The APC/C Co-activator Cdh1 Promotes BRSK2 Degradation

**Figure 4:**
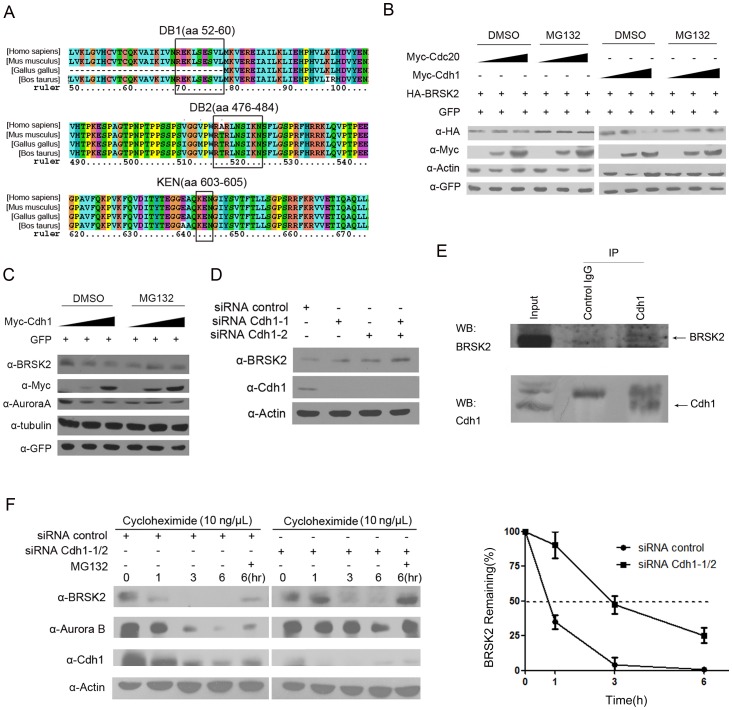
The APC/C co-activator Cdh1 promotes BRSK2 degradation. (A) Sequence alignment of BRSK2 orthologs from different species, including *Homo sapiens*, *Mus musculus, Gallus gallus* and *Bos taurus*. The two conserved D boxes and the KEN box are outlined. (B) HA-BRSK2 was co-transfected with increasing amounts of Myc-Cdh1 or Myc-Cdc20 into HeLa cells. MG132 (20 µM) was added 6 h before harvest. Cells were harvested, and exogenous BRSK2 protein levels were analyzed. (C) Increasing amounts of Myc-Cdh1 were transfected into HeLa cells that were treated with MG132 similarly to (B). Cells were harvested, and endogenous BRSK2 protein levels were analyzed. pEGFP-C1 co-transfection in (B) and (C) served as a control for transfection efficiency, and GFP levels were assessed using anti-GFP antibodies. (D) HeLa cells were transiently transfected with one or both of two Cdh1-specific siRNAs or control siRNA. Forty-eight hours after transfection, BRSK2 and Cdh1 protein levels were analyzed by Western blotting (WB). (E) Expression of Cdh1 was knocked down by co-transfection of the two Cdh1-specific siRNAs. HeLa cells were treated with 10 ng/µL cycloheximide for the indicated times. HeLa cells treated for 6 h were also treated with (+) or without (−) MG132. Whole-cell extracts were Western blotted (WB) with the indicated antibodies. The graph shows the quantification of BRSK2 levels using actin as a control. The error bars represent the mean ± S.D. from three independent experiments. (F) HEK293T cells were arrested by a nocodazole block and lysed, and then, the cell lysates were incubated with Protein A/G-Sepharose conjugated with either control IgG or Cdh1 antibody. The immunoprecipitates were washed, and bound proteins were resolved on an SDS-PAGE gel followed by Western blotting (WB).

APC/C substrates generally contain degrons such as D boxes or KEN boxes, which are required for their degradation. We wanted to determine whether the BRSK2 protein also possessed these elements. We carefully inspected the sequence of BRSK2 and identified a putative KEN box corresponding to the C-terminal residues 603–605 in human BRSK2. We also found two putative D boxes in human BRSK2 at residues 52–60 and 476–484 (Fig. 4A). Mouse, chicken and cow BRSK2 proteins all contain homologous D box motifs corresponding to residues 52–60 (named DB1) and 476–484 (named DB2) of human BRSK2, with the exception that mouse BRSK2 lacks DB1 (Fig. 4A).

Additionally, Hsl1 (BRSK2 homolog in *S. Cerevisiae*) protein is a substrate of APC/C^Cdh1^ [33], [34]. Given the findings described above, we investigated whether BRSK2 is a target of APC/C^Cdh1^. We transfected HeLa cells with the same amount of HA-BRSK2 and increasing amounts of Myc-Cdh1 or Myc-Cdc20. As expected, overexpression of Cdh1 greatly reduced the protein levels of HA-BRSK2, while no reduction of protein level of HA-BRSK2 by Myc-Cdc20 was detected (Fig. 4B). To verify whether Cdh1 promotes the degradation of BRSK2, we examined endogenous BRSK2 protein levels when increasing amounts of Myc-Cdh1 were transfected. Our results indicated that a reduction in endogenous BRSK2 levels could be caused by Myc-Cdh1 transfection (Fig. 4C). These results indicated that BRSK2 is a substrate for APC/C^Cdh1^.

We next showed that Cdh1 is required for the degradation of BRSK2 *in vivo*. We knocked down endogenous Cdh1 using either or both of two specific siRNAs (siRNA Cdh1-1 and siRNA Cdh1-2) and determined whether the BRSK2 protein levels changed in HeLa cells. The knock down of Cdh1 resulted in an increase in endogenous BRSK2 protein levels (Fig. 4D). Furthermore, we observed that co-transfection of both Cdh1 siRNAs produced a stronger knock-down effect; therefore, we performed the following experiment using the co-transfection of both siRNAs. Cells co-transfected with both siRNAs and treated with cycloheximide were examined at various time points to determine the half-life of BRSK2 *in vivo*. Knock down of Cdh1 resulted in a significant increase in the stability of BRSK2, increasing the half-life of BRSK2 to nearly 3 h, in contrast to 1 h in the control (Fig. 4E). We again confirmed that BRSK2 is degraded through the proteasomal pathway by clearly observing that BRSK2 degradation was inhibited in the cells treated with MG132 for 6 h (Fig. 4E).

To confirm that BRSK2 is a target of APC/C^Cdh1^ *in vivo*, we examined whether Cdh1 interacts BRSK2 in mitotic cells because the BRSK2 protein level is extremely low in G1. HEK293T cells were synchronized at G2/M through treatment with nocodazole for 20 h and then preincubated with MG132 for 6 h before harvest. Cdh1 was immunoprecipitated from the cell lysates using anti-Cdh1 antibody, and BRSK2 was examined using anti-BRSK2 antibody. The complexes immunoprecipitated by Cdh1 contained BRSK2; however, BRSK2 was not detected in complexes immunoprecipitated by the IgG control (Fig. 4F). These data indicated that BRSK2 and Cdh1 can form a complex during mitosis progression.

Thus, BRSK2 stability is controlled by the APC/C^Cdh1^-ubiquitin-proteasome pathway *in vivo*.

### KEN Box is Required for BRSK2 Degradation

**Figure 5:**
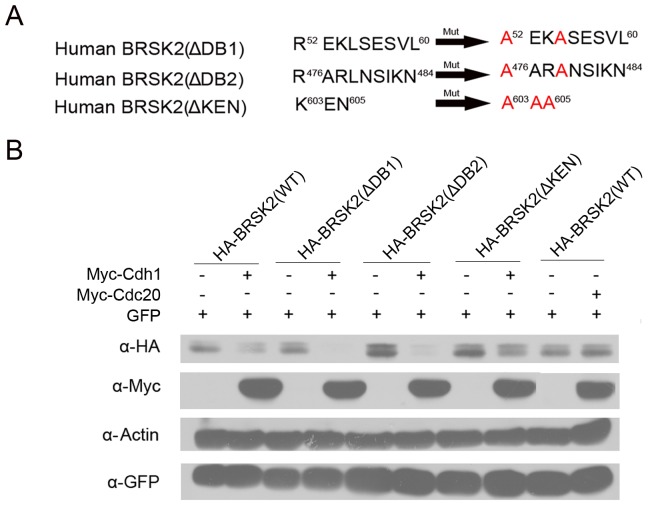
The KEN box is essential for BRSK2 degradation. The same amount of wild type or various mutant HA-BRSK2 expression constructs were co-transfected with (–) or without (+) Myc-Cdh1 or Myc-Cdc20 plasmid into HeLa cells. The protein levels of BRSK2 and Cdh1 were determined by Western blotting (WB) with anti-HA and anti-Myc antibodies, respectively. The pEGFP-C1 expression construct was included as a control for transfection efficiency.

Cdh1 targets APC/C^Cdh1^ substrates for degradation and requires the recognition of either a KEN or D box in the substrates [28]. To determine whether these motifs are important for BRSK2 degradation, we first mutated the 1^st^ and 3^rd^ amino acids to alanine in either DB1 or DB2, which we named HA-BRSK2(ΔDB1) and HA-BRSK2(ΔDB2), respectively. We also mutated the 1^st^, 2^nd^, and 3^rd^ amino acids to alanine in KEN box, which we named HA-BRSK2(ΔKEN) (Fig. 5A). HA-BRSK2 mutant or wild type (WT) constructs were transfected into HeLa cells with or without the Myc-Cdh1 plasmid, and the BRSK2 protein levels were examined by Western blotting. Cdh1 overexpression caused a reduction in both HA-BRSK2(ΔDB1) and HA-BRSK2(ΔDB2) levels that was similar to the reduction observed for HA-BRSK2(WT) (Fig. 5B), which suggests that both mutants are substrates of APC/C^Cdh1^ and that DB1 and DB2 are not essential for BRSK2 degradation. In contrast, it appeared that the HA-BRSK2(ΔKEN) levels were independent of Cdh1 overexpression in HeLa cells (Fig. 5B). To verify that Cdh1, rather than Cdc20, mediates the degradation of BRSK2, HA-BRSK2(WT) was transfected into HeLa cells with or without the Myc-Cdc20 plasmid. Again, we observed that Cdc20 had no effect on BRSK2 degradation (Fig. 5B). Thus, the KEN box of BRSK2 serves as a degradation signal recognized by the APC/C activator, Cdh1.

### Overexpression of BRSK2 Increases the G2/M Populations

**Figure 6:**
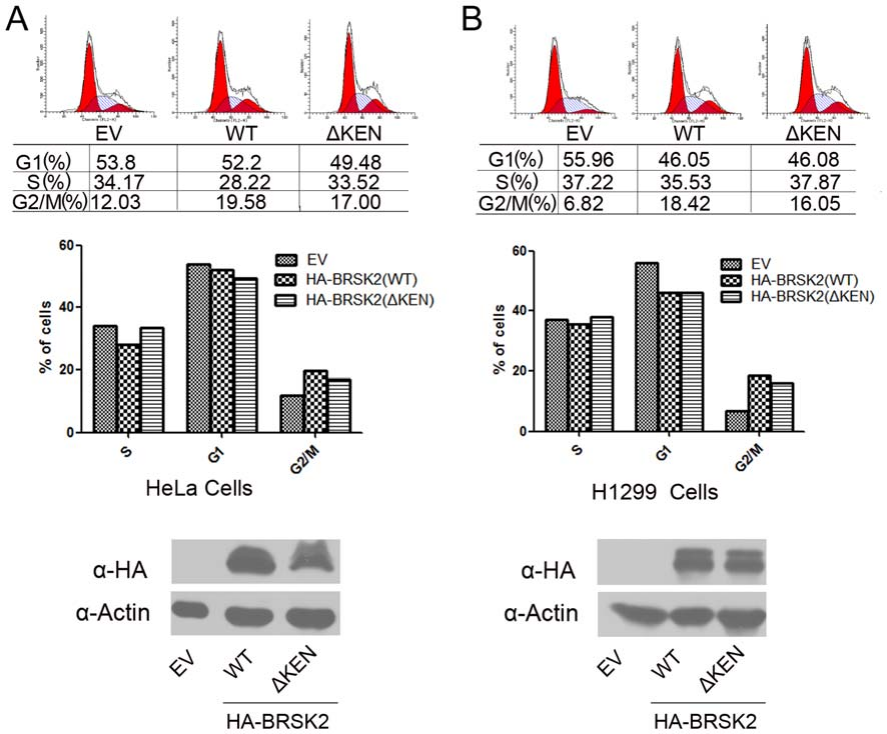
Over-expression of BRSK2 increases the population of cells in G2/M. Control, BRSK2(WT) or BRSK2(ΔKEN) plasmids were transfected with pBB14-GFP plasmid into HeLa cells and H1299 cells. Cells were harvested for flow cytometry analysis and Western blotting (WB) analysis using indicated antibody. The pBB14-GFP expression construct served as a positive transfection marker for flow cytometry analysis.

Previous studies suggested SAD kinases may be involved in regulating cell cycle progression. To explore the role of BRSK2 in cell cycle regulation, HA-BRSK2(WT) or HA-BRSK2(ΔKEN) plasmids were transfected with pBB14-GFP plasmid into HeLa cells. We observed an increase in the number of HeLa cells transfected with either HA-BRSK2(WT) or HA-BRKS2(ΔKEN) plasmids that were in G2/M (19.58%, 17.00%) compared to control (12.03%) (Fig. 6A). To further confirm the cell cycle regulatory function of BRSK2, we examined the G2/M populations in H1299 cells. Similar to the results observed with HeLa cells, the population cells in G2/M was 18.42%, 16.05% and 6.82%, for HA-BRSK2(WT), HA-BRSK2(ΔKEN) and the control, respectively (Fig. 6B). Our results imply that BRSK2 regulates mitosis.

## Discussion

BRSK2 was originally identified as a gene expressed specifically in the brain [2], but recent studies showed that BRSK2 is also expressed in some tumor cell lines, such as HeLa and Panc-1 [3], which implies that BRSK2 might have complex and important biological functions related to tumor cell growth. Although the phosphorylation-based mechanism regulating BRSK2 kinase activity has been well characterized, the detailed molecular mechanism regulating BRSK2 protein levels is still unknown. Each member of the AMPK family has an ubiquitin-associated domain (UBA domain) or a similar structure. Studies have shown that this UBA domain of AMPK-RKs is phosphorylated by the upstream kinase LKB1 and tightly regulates kinase activity but has no effect on protein stability [19]. Intriguingly, the polyubiquitination of AMPK and several other AMPK-RKs through atypical lysine29- and lysine33-linked chains was reported recently [18]. Another elegant study found that the E3 ubiquitin ligase Cidea (cell death inducing DFFA-like effector a) governs the ubiquitination and degradation of AMPK [20]. However, lysine29- and lysine33-linked polyubiquitin chains are generally not involved in the proteasomal degradation system; therefore, determining which type(s) of lysine-linked polyubiquitin chains are involved and to which region of BRSK2 the chains bind requires further investigation. We also cannot exclude the possibility that BRSK2 destruction is mediated by other degradation systems. Thus, further studies are needed to define the mechanism of BRSK2 post-translational regulation.

Previous studies proposed that BRSK1/2 plays an important role in the distribution of synaptic vesicles, synapse development and neuronal polarization. Emerging data on the cellular function of BRSK1/2 suggest that it regulates the cell cycle by interacting with several key cell cycle regulators. BRSK1 (SADB) phosphorylates the cell cycle regulatory kinases Wee1 and Cdc25B/C [8], and another study proposed that BRSK2 (SADA) also regulates the Wee1 activity though phosphorylation [14]. Consistent with these data, we showed that BRSK2 controls mitosis through the phosphorylation of Cdc25C (Wang et al, submitted). Over the past few decades, studies of BRSK2 have revealed its importance in physiological function, although the exact molecular mechanisms underlying the regulation its activity are still not well understood. BRSK1 (SADB) kinase activity fluctuates during cell cycle progression in response to phosphorylation, but little is known about the mechanism of phosphorylation regulation [17]. It was reported that PP2C significantly inhibits BRSK2 activity *in vitro* [2]. In this paper, we demonstrated that APC/C^Cdh1^ targets BRSK2 for degradation through the ubiquitin-proteasome pathway. We speculated that the mitotic degradation of BRSK2 by APC/C^Cdh1^ contributes to the regulation of kinase activity by controlling protein levels. BRSK2 is unstable during cell cycle progression and declines to a low, but still detectable level in G1 phase (Fig. 2A). We suggest that BRSK2 might be a multifunctional protein serving several molecular functions in addition to regulating mitosis. BRSK2 was identified as a novel centrosome co-localized protein, implying that BRSK2 regulates the cell cycle through interaction with γ-tubulin or other mitosis regulators, such as polo-like kinase [17], [35]. Taken together, these data imply that BRSK2 regulates cell cycle progression through an intricate signaling network.

Another study suggested that APC/C^Cdh1^ plays a critical role in the control of axonal growth and patterning in mammalian brain [36]. APC/C^Cdh1^ targets the transcriptional corepressor SnoN for timely degradation to control the axonal morphogenesis [37]. The importance of BRSK2 regulation of axonal development has been well characterized. It will be interesting to determine whether BRSK2 controls axonal development through the APC/C^Cdh1^-ubiquitin-proteasome mediated pathway and if BRSK1 (SADB) also undergoes degradation in a pattern similar to BRSK2.

In summary, we described a molecular mechanism for the regulation of BRSK2 mitotic expression. Additionally, we presented several lines of evidence to suggest that APC/C^Cdh1^ is responsible for the ubiquitination and mitotic degradation of BRSK2. First, BRSK2 contains a highly conserved degron (KEN box) that can be specifically recognized by Cdh1 (Fig. 4A). Second, BRSK2 degradation can be inhibited by treatment of the proteasome inhibitor MG132 *in vivo* (Fig. 3A). Third, Cdh1 accelerates the polyubiquitination and degradation of BRSK2 (Fig. 4D, E and G). Finally, the KEN box is essential for APC/C^Cdh1^ mediated BRSK2 degradation (Fig. 5B). Thus, we conclude that BRSK2 is a novel substrate of APC/C^Cdh1^ during the cell cycle.
